# The effect of insulin and kruppel like factor 10 on osteoblasts in the dental implant osseointegration in diabetes mellitus patients

**DOI:** 10.1080/21655979.2022.2084534

**Published:** 2022-06-22

**Authors:** Chen Sheng, Yalin Guo, Wenjie Hou, Haobin Chen, Hongchen Liu, Lin Wang

**Affiliations:** a Department of Stomatology, Medical School of Chinese PLA, Beijing, China; b Department of Osteology, Medical School of Chinese PLA, Beijing, China; c Department of Stomatology, The First Medical Centre, Chinese PLA General Hospital, Beijing, China

**Keywords:** Diabetes mellitus, dental implant, insulin, KLF10, osteoblasts, bone metabolism, AKT, NF-κB

## Abstract

Diabetes mellitus, metabolic disease, is characterized by chronic hyperglycemia. Patients with diabetes mellitus are susceptible to infection and therefore have a higher prevalence and progression rate of periodontal disease. We aimed to study the effect of insulin and kruppel like factor 10 (KLF10) on osteoblasts proliferation and differentiation, and expression of bone metabolism-related molecules and related signaling pathway molecules of AKT serine/threonine kinase 1 (AKT) and nuclear factor kappa B subunit 1 (NF-κB) through in vitro experiments, which can provide theoretical basis for the dental implant osseointegration in diabetic patients. The osteoblasts (hFOB 1.19 cells) were subdivided into KLF10 gene over expression group, KLF10 gene knockdown group, and KLF10 gene knockdown + insulin treatment group. CCK-8 and ELISA were, respectively, used for analysis of cell proliferation and differentiation. In vitro experiments were applied to detect the mRNA and protein expression of bone metabolism-related molecules, respectively. GSE178351 dataset and GSE156993 dataset were utilized to explore the expression of KLF10 in periodontitis. In osteoblasts, insulin treatment increased the expression of KLF10. Insulin and KLF10 could reduce the proliferation and differentiation of osteoblasts. Knockdown of KLF10 could increase the expression of bone metabolism-related molecules and activate AKT and NF-κB pathways, whereas insulin reversed this effect. KLF10 was up-regulated in both patients with periodontitis and type 2 diabetes mellitus with periodontitis. It is assumed that knockdown of KLF10 in insulin resistance may promote osteoblasts differentiation and dental implant osseointegration in diabetic patients.

## Graphical Abstract

**Figure:**
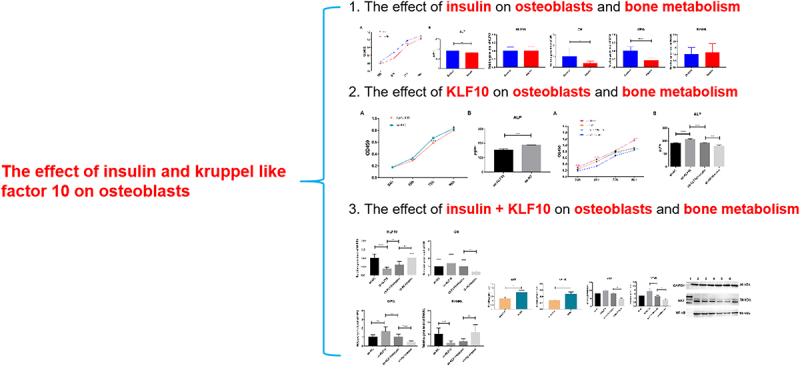

## Highlights

- In osteoblasts, insulin treatment increased the expression of KLF10.
- Insulin and KLF10 could reduce the proliferation and differentiation of osteoblasts.
- Knockdown of KLF10 affects bone metabolism-related molecules and pathways.
- KLF10 was up-regulated in patients with type 2 diabetes mellitus with periodontitis.
- Knockdown of KLF10 promotes dental implant osseointegration in diabetic patients.

## Introduction

Diabetes mellitus is caused by an insufficient action or decreased production or of insulin [1]. The decrease in gene expression of osteoblast differentiation and diminished extracellular matrix production is found in diabetes mellitus [2–4]. In addition, the hyperglycemic state is related to numbers of complications, leading to periodontitis and impaired wound healing [5,6]. Implant-supported prostheses have become the major choice for restoring oral function in patients with dental disease. However, diabetes mellitus is considered the relative contraindication for implanting therapy. It is assumed that poor glycemic control is directly associated with short-term-impairment implant stabilization [7]. In addition, changes in implant stability were consistent with resorptive and formative osseous healing after implant placement [8].

Recently, identifying the molecular mechanisms underlying bone tissue remodeling disorders around dental implants in patients with diabetes mellitus is the focus of active research. Clinically, insulin administration remains the major method of diabetes mellitus management [9]. Insulin therapy can ameliorate bone regeneration of implants for diabetics [10–12]. But, the concrete effects are still debatable. In addition, insulin alone is inadequate to reverse all the adverse effects of type 2 diabetes (T2DM) on implant osseointegration [13]. Hence, it is needed to find new treatment modalities to enhance dental implant osseointegration in patients with T2DM.

Through high-throughput sequencing, our previous research found that kruppel like factor 10 (KLF10) was up-regulated after insulin treatment and associated with new bone formation [14]. However, the specific regulation mechanism remains unclear. KLF10 (also called TIEG) is originally identified as a transforming growth factor (TGF)-β-inducible early gene 1 in osteoblasts, which play important roles in bone metabolism [15–19]. KLF10 plays an important role in proliferation and inflammatory response [20]. It is found that KLF10 is down-regulated in the osteoarthritic knee [21]. It is shown that mRNA or protein level of KLF10 is significantly increased in urinary exosomes of patients with diabetic nephropathy [22]. Yerges LM et al. found that the single nucleotide polymorphisms in the KLF10 gene was related to decreased volumetric bone mineral density at the femoral neck [23]. In addition, KLF10 is involved in tooth development and promotes odontoblast differentiation by up-regulating the transcription of odontoblast marker genes [24]. Up to now, the effect of insulin and KLF10 on osteoblasts proliferation and differentiation, expression of bone metabolism-related molecules and related signaling pathway molecules of AKT serine/threonine kinase 1 (AKT) and nuclear factor kappa B subunit 1 (NF-κB) level on dental implant osseointegration in patients with diabetes mellitus is still unclear. In view of this, we assume that insulin and KLF10 may play roles in dental implant osseointegration. In this study, we aimed to study the effect of insulin and KLF10 on osteoblast bone metabolism through in vitro experiments. Our study may provide a new field in understanding the pathological mechanism of KLF10 in osteoblasts differentiation and dental implant osseointegration in diabetic patients.

## Materials and methods

### Cell culture

The osteoblasts (hFOB 1.19 cells) were cultured in the cell plates with complete medium of DMEM/F12 supplemented with 10% of fetal bovine serum (FBS), 1% of penicillin/streptomycin and 0.3 mg/mL of G418 (a reagent used to maintain cell stability after transfection) in a 5% CO_2_ humidified atmosphere incubator at 37°C. Cells were divided into the following six groups: sh-KLF10 group (KLF10 gene knockdown), sh-NC group (control group of KLF10 gene knockdown), sh-KLF10 + 200 ng/ml of insulin treatment group, sh-NC+200 ng/ml of insulin treatment group, oe-KLF10 group (KLF10 gene over expression), and oe-NC group (control group of KLF10 gene over expression). The experimental method was referred from a previous study [25]. One day before transfection, 0.5–2 × 10^5^ cells were inoculated in 1500ul nonresistant medium. The degree of cell fusion was 50% at transfection. SiRNA/plasmid transfected cells were diluted with 250ul Opti-medium. The cells were gently aspirated and mixed 3–5 times. The transfection reagent was gently reversed and mixed. A total of 6ul LipofectamineTM 2000 was diluted with 250uL opti-medium, then gently beaten for 3–5 times, and stood still at room temperature for 5 min. The transfection reagent and SiRNA/plasmid diluent (100 nM) were mixed, and gently blew 3–5 times. The mixture was left at room temperature for 20 min. The transfection mixture was added to the 6-well cell plate. Cell culture plates were placed in 37%, 5°C CO_2_ incubator for 24–48 h. After transfection for 4–6 h, the culture medium could be replaced by fresh medium.

### Cell proliferation analysis

Cell proliferative capacity of the osteoblasts was detected by CCK-8 cell proliferation analysis according to previous research [26]. 100 μl of cell suspension (about 1000–3000 cells) was added to each well of 96-well plate. 10 μl of CCK-8 solution was added to each well. The wells in blank cell culture with CCK-8 solution were used as blank control. Cells were incubated in the cell culture box for 0.5–4 h. The absorbance of the 96-well plate was measured at 450 nm in a microplate reader for further statistical analysis of data.

### ELISA detection

Alkaline phosphatase (ALP) is a marker enzyme for osteoblasts differentiation and maturation. ALP activity in osteoblasts was detected by ELISA method [27]. The protein antibody was diluted with 0.05 M carbonate coated buffer (pH of 9.0) to protein content of 1–10 μg/ml. 0.1 ml of antibody dilution was added to the reaction well of each polystyrene plate overnight at 4°C. The next day, the solution in the well was discarded. The antibody dilution was washed with washing buffer for 3 times. 0.1 ml of antibody diluted sample was added to the coated reaction well and incubated at 37°C for 1 h. In addition, blank well, negative control well and positive control well was set. 0.1 ml of freshly diluted HRP-labeled antibody was added into each reaction well and incubated at 37°C for 0.5–1 h. 0.1 ml of temporary tetramethyl benzidine (TMB) substrate solution was added into each reaction well at 37°C for 10–30 min. 0.05 ml of 2 M sulfuric acid was added into each reaction well to stop the reaction. The result was analyzed on the ELISA detector at 450 nm to detect the OD value of each well.

### Quantitative real‐time polymerase chain reaction (QRT-PCR)

Based on detailed methods in previous report [28], after treatment with trypsin, the cell solution was transferred to a RNAse-free centrifuge tube, centrifuged at 300 × g for 5 min. Cell precipitate was collected to remove supernatant and incubated with TRIzol reagent for 5 min. Cells were incubated with Trichloromethane (200 μl/1 ml TRIzol reagent) and shook vigorously for 45 s and stand at room temperature for 3 min. Then, cells were centrifuged at 12,000 rpm at 4°C for 10 min. The supernatant was transferred to a new centrifugal tube, and added equal volume of 70% of ethanol, and mixed inversely. All the solution was stepped into the adsorption column and centrifuged at 12,000 rpm for 20 s. 700 μl of Buffer RW1 was added to the adsorption column and centrifuged at 12,000 rpm for 20 s. 500 μl of Buffer RW2 was added into the adsorption column containing anhydrous ethanol and centrifuged at 12,000 rpm for 20 s. The adsorption column was placed at room temperature for several minutes and dried thoroughly. The adsorption column was filled into a new RNAse-free centrifuge tube. 30–50 μl of RNAse-free water was added to the middle part of the adsorption column, placed at room temperature for 1 min, and centrifuged at 12,000 rpm for 1 min. The RNA solution was collected and stored at −70°C to prevent degradation. 2 μg of RNA was applied to synthesize cDNA. Then, quantitative real‐time polymerase chain reaction was performed. The annealing temperature is 60°C. All reactions were performed in triplicate. Relative gene expression was analyzed by 2^−ΔΔCT^ method. Gene primers of the qRT-PCR analyses are listed in Table 1.

**Table 1.** The gene primers of the qRT-PCR analyses.

Genes	Upstream primer sequence	Downstream primer sequence
GAPDH	AGGGGCCATCCACAGTCTTC	AGGGGCCATCCACAGTCTTC
KLF10	CGCTGTCCATTGCAGCTTAC	TGCATGATGCCTTCGTGTTG
ON	CAAGAAGCCCTGCCTGATGA	TCTTCGGTTTCCTCTGCACC
OPG	CCTCTGTGAAAACAGCGTGC	AGGTGTCTTGGTCGCCATTT
RANKL	CACAGCACATCAGAGCAGAG	TACCAAGAGGACAGACTCACTT

### Western Blot test

Detailed experimental methods of Western Blot were based on previous study [29]. Cells were rinsed in PBS for 2–3 times. The remaining fluid was drained thoroughly for the last time. An appropriate volume of RIPA containing protease inhibitor was added in the culture plate and bottle and mixed for 3–5 minutes. The culture plate and bottle were shaken repeatedly. The cells were scraped off with a cell scraper and collected into a 1.5 ml centrifuge tube and treated with ice bath for 30 min. Pipette was used to blow cells repeatedly to ensure complete cell lysis. Cells were centrifuged at 12,000 rpm at 4°C for 10 min to collect the total protein solution. Protein concentration was measured for SDS-PAGE electrophoresis, transmembrane of protein and immunoreactions. In the immunoreactions, antibodies of AKT (Abcam, 1:5000), NF-κB (Abcam, 1:1000) and GAPDH (Abclonal, 1:50,000) were used. The film was placed in the imager for exposure.

### Expression analysis of KLF10 in patients with periodontitis and type 2 diabetes mellitus with periodontitis

In order to further investigate the expression of KLF10 in periodontitis (a diabetes mellitus related periodontal disease), GSE178351 dataset (involving soft tissue around the implant samples from four peri-implantitis patients and three healthy individuals) and GSE156993 dataset (involving peripheral blood mononuclear cell samples from five poorly controlled T2DM with periodontitis and six healthy individuals) were used for analysis. The result was presented as a box plot.

In this study, we aimed to study the effect of insulin and KLF10 on osteoblasts proliferation and differentiation, and expression of bone metabolism-related molecules and related signaling pathway molecules of AKT and NF-κB through in vitro experiments. Knockdown of KLF10 could increase the expression of bone metabolism-related molecules and activate AKT and NF-κB pathways, whereas insulin reversed this effect. Our study may provide a theoretical basis for the dental implant osseointegration in diabetic patients

## Results

### The effect of insulin and KLF10 on proliferation and differentiation of osteoblasts

**Figure 1.**
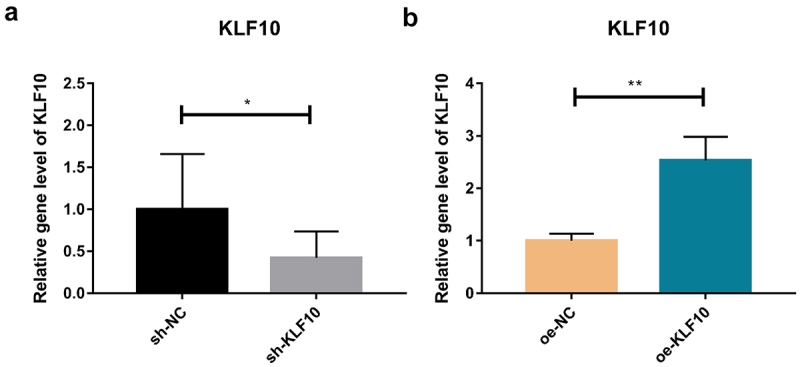
Efficiency detection of KLF10 interference (a) and over expression (b) in osteoblasts by quantitative real‐time polymerase chain reaction.

**Figure 2.**
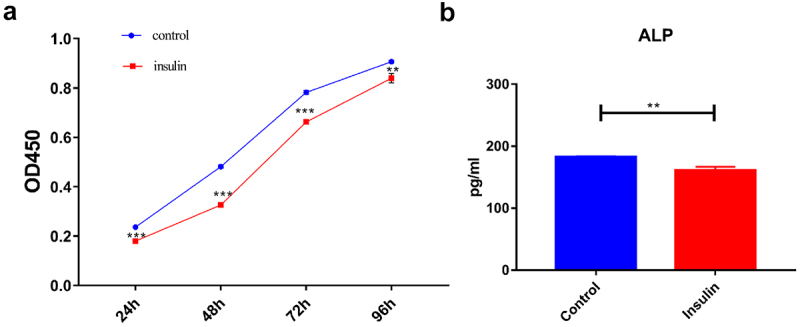
The effect of insulin on proliferation (a) and differentiation (b) of osteoblasts.

**Figure 3.**
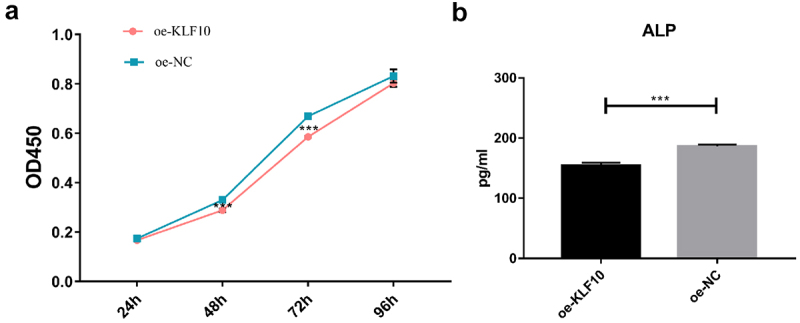
The effect of KLF10 over expression on proliferation (a) and differentiation (b) of osteoblasts.

**Figure 4.**
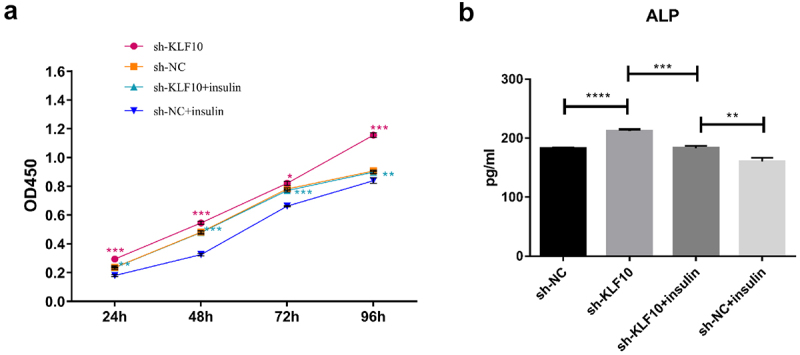
The effect of KLF10 knockdown and insulin on proliferation (a) and differentiation (b) of osteoblasts.

The KLF10 gene interference and over expression experiments were performed successfully (Figure 1(a,b)). After insulin treatment, the proliferation ability of osteoblasts was weakened and the number of osteoblasts was significantly reduced at 24, 48, 72, and 96 h (Figure 2(a)). At the same time, ALP activity was remarkably decreased (Figure 2(b)). Over expression of KLF10 significantly decreased the proliferation rate (Figure 3(a)) and ALP activity of osteoblasts (Figure 3(b)). When KLF10 was knocked down, the proliferation of osteoblasts was significantly enhanced; the addition of insulin remarkably inhibited the proliferation of osteoblasts (Figure 4(a)). In addition, knockdown of KLF10 significantly increased the activity of ALP, while insulin remarkably inhibited the activity (Figure 4(b)). It is indicated that insulin and KLF10 could reduce the proliferation and differentiation of osteoblasts and promote bone resorption in osteoblasts.

*p < 0.05; **p < 0.01. A total of three biological and technical replicates were performed. The T-test was used for statistical analysis.

**p < 0.01; ***p < 0.001. A total of three biological and technical replicates were performed. The T-test was used for statistical analysis.

***p < 0.001. A total of three biological and technical replicates were performed. The T-test was used for statistical analysis.

*p < 0.05; **p < 0.01; ***p < 0.001; ****p < 0.0001. A total of three biological and technical replicates were performed. The T-test was used for statistical analysis.

### The effect of insulin and KLF10 on bone metabolism-related molecules

**Figure 5.**
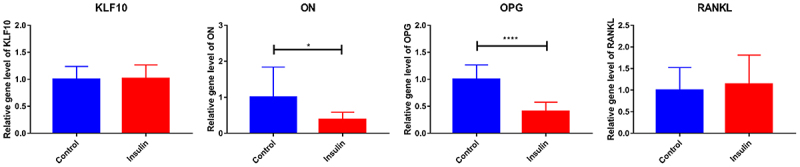
The effect of insulin on mRNA expression of KLF10, ON, OPG and RANKL.

**Figure 6.**
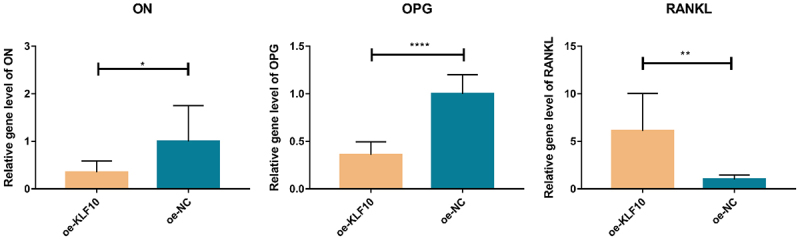
The effect of KLF10 over expression on mRNA expression of ON, OPG and RANKL.

**Figure 7.**
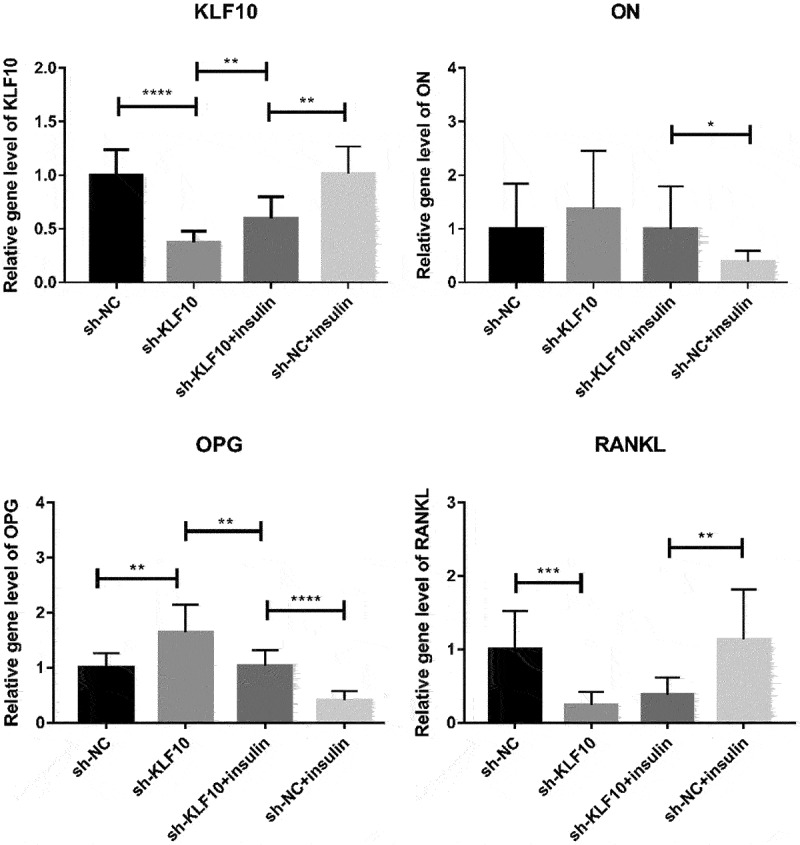
The effect of KLF10 knockdown and insulin on mRNA expression of KLF10, ON, OPG, and RANKL.

After insulin treatment, the expressions of KLF10, secreted protein acidic and rich in cysteine (SPARC, also known as osteonectin (ON), osteoprotegerin (OPG) and receptor activator of nuclear factor kappa-Β ligand (RANKL) were detected by QRT-PCR. KLF10 and RANKL tended to be up-regulated with no significant difference. ON and OPG were significantly down-regulated (Figure 5). Over expression of KLF10 significantly decrease expression of ON and OPG and remarkable increase expression of RANKL (Figure 6). The effect of KLF10 knockdown and insulin on mRNA expression of KLF10, ON, OPG, and RANKL is illustrated in Figure 7. Insulin significantly increased the expression of KLF10 after knockdown of KLF10. After knockdown of KLF10, ON was dramatically up-regulated in the presence of insulin. After knockdown of KLF10, OPG expression was significantly up-regulated and remarkably down-regulated after insulin treatment. Moreover, the expression of OPG was significantly increased in the presence of insulin after knockdown of KLF10. After KLF10 was knocked down, RANKL expression was significantly down-regulated. However, after insulin treatment, RANKL expression was reversed but not significant. It is noted that RANKL was significantly up-regulated in the presence of insulin after knockdown of KLF10. This suggested that insulin can increase the expression of KLF10, and knockdown of KLF10 may promote bone metabolism.

*p < 0.05; ****p < 0.0001. A total of three biological and technical replicates were performed. The T-test was used for statistical analysis.

*p < 0.05; **p < 0.01; ****p < 0.0001. A total of three biological and technical replicates were performed. The T-test was used for statistical analysis.

*p < 0.05; **p < 0.01; ****p < 0.0001. A total of three biological and technical replicates were performed. The T-test was used for statistical analysis.

### The effect of insulin and KLF10 on signaling pathway of AKT and NF-κB

**Figure 8.**
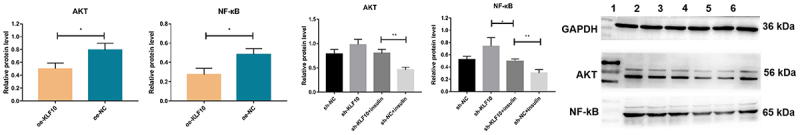
The effect of insulin and KLF10 on protein expression of AKT and NF-kB.

In order to study the effect of insulin and KLF10 on the signaling pathway of AKT (total AKT) and NF-κB, Western Blot was performed to detect the protein expression of AKT and NF-κB (Figure 8). The expression of AKT and NF-κB was significantly decreased after over expression of KLF10. When KLF10 was knocked down, the expression of AKT and NF-κB was significantly up-regulated. The addition of insulin reversed the expression trend of these factors. This indicated that knockdown of KLF10 in osteoblasts could activate AKT and NF-κB pathways, whereas insulin reversed this effect. It is assumed that knockdown of KLF10 in insulin resistance may promote osteoblast differentiation and bone healing.

*p < 0.05; **p < 0.01. 1, 2, 3, 4, 5, and 6 represents sh-KLF10 group, sh-NC group, sh-KLF10 + insulin treatment group, sh-NC + insulin treatment group, oe-KLF10 group and oe-NC group, respectively. A total of three biological and technical replicates were performed. The T-test was used for statistical analysis.

### Expression analysis of KLF10 in patients with periodontitis and type 2 diabetes mellitus with periodontitis

**Figure 9.**
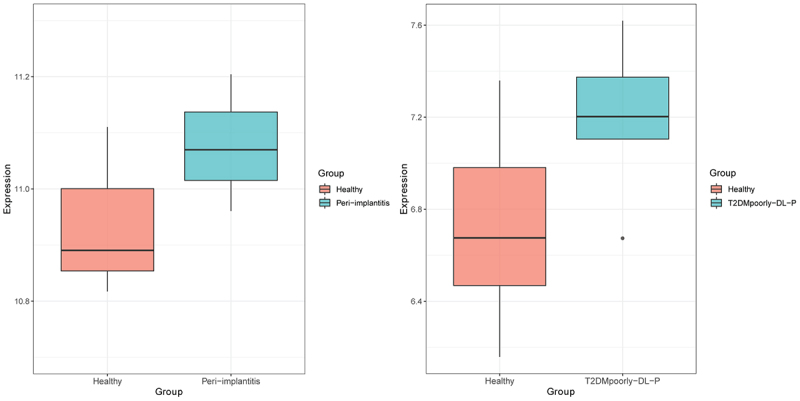
Expression box plot of KLF10 in patients with periodontitis and type 2 diabetes mellitus with periodontitis.

GSE178351 dataset (involving four peri-implantitis patients and three healthy people) dataset and GSE156993 dataset (involving five poorly controlled T2DM with periodontitis and six healthy individuals) were used to analyze the expression level of KLF10. KLF10 was up-regulated in both patients with periodontitis and Type 2 diabetes mellitus with periodontitis (Figure 9). This indicated that high expression of KLF10 may be involved in the process of periodontitis of type 2 diabetes mellitus.

## Discussion

In this study, we found that insulin and KLF10 reduced the proliferation and differentiation of osteoblasts. Moreover, knockdown of KLF10 increased the expression of bone metabolism-related molecules and activated AKT and NF-κB pathways, whereas insulin reversed this effect. It is suggested that knockdown of KLF10 in insulin resistance could promote osteoblast differentiation.

Osteoblasts first synthesize collagen-I, osteocalcin, osteopontin, and other extracellular matrix. In addition, the matrix vesicles release calcium ions and ALP. Calcium ions precipitate on the collagen filaments to complete the matrix mineralization process and eventually form bone tissue. ON (also called SPARC) is associated with remodeling (bone) and wound healing [30,31]. In wound repair, ON plays an important role [32]. The On-null mice develop osteopenia around 2.5 months of age in bones [30,33]. In addition, ON is related to fast insulin levels [34]. Increasing doses of glucose can decrease protein expression of ON [34]. OPG and RANKL play important roles in bone metabolism. During the differentiation of bone marrow mesenchymal stem cells into osteoblasts, OPG secretion increased rapidly and OPGL decreased significantly. The increase of OPG/OPGL ratio can inhibit osteoclast formation and decrease bone resorption [35]. The OPG/RANK/RANKL system could have the potential role in diabetes mellitus. Blocking the pathway will prevent the development of diabetes mellitus [36]. The proliferation and osteogenic differentiation abilities of osteoblasts is weaker in diabetes mellitus patients [37]. For most patients with diabetes mellitus, despite improvements in insulin replacement therapy, reduced bone mass and low bone mineral density are observed [38–41]. It is reported that insulin signaling in osteoblasts promotes the differentiation of osteoclasts [42]. In this study, we found that after insulin treatment, the proliferation ability of osteoblasts was weakened and the number of osteoblasts was significantly decreased at 24, 48, 72, and 96 h. The differentiation activity of osteoblasts was remarkably reduced. In addition, after insulin treatment, ON and OPG were significantly down-regulated. This indicated that insulin could play a dual role in bone metabolism.

KLF10 is associated with some biological processes, including cell differentiation and glucose metabolism [15,16,18,43,44]. In diabetic mice, the expression of Klf10 is increased in the liver and knockdown of Klf10 can decrease blood glucose levels and improve glucose tolerance [18]. It is found that KLF10 participated in the insulin-signaling pathway [45]. It is noted that the variant of the KLF10 gene contributes to the risk of type-2 diabetes mellitus [46]. In this study, we found that insulin could increase the expression of KLF10 in osteoblasts. This indicated that KLF10 may be involved in glucose metabolism under the regulation of insulin. In addition, KLF10 is identified in clinical studies as a gene whose altered expression levels or allelic variations are linked to decreased bone mass and osteoporosis [47]. Chen Z et al. found that KLF10 is involved in the dental cell proliferation and differentiation and tooth development induced by bone morphogenetic protein 2 (BMP2) signaling pathway [24]. KLF10 can directly bind to GC-rich elements in the OPG promoter to suppress gene transcription [48]. It is shown that the reduction in the ability of KLF10 knockout osteoblasts for osteoclast differentiation [49]. Increased OPG and decreased RANKL expression are found in KLF10 knockout calvarial osteoblasts, suggesting potential roles for KLF10 in regulating the expression of these genes [48]. Herein, we found that KLF10 reduced the proliferation and differentiation of osteoblasts. Moreover, over expression of KLF10 significantly decreases expression of ON and OPG and remarkably increases expression of RANKL. After knockdown of KLF10, ON and OPG were significantly up-regulated, RANKL was significantly down-regulated. However, insulin treatment reversed this effect. This indicated that insulin can increase the expression of KLF10, and knockdown of KLF10 may promote bone metabolism. In addition, we found that KLF10 was up-regulated in both patients with periodontitis and type 2 diabetes mellitus with periodontitis. This indicated that high expression of KLF10 may be associated with the process of periodontitis of type 2 diabetes mellitus.

The PI3K-AKT-mTOR (a classical pathway in response to insulin signaling) promotes the proliferation and differentiation of osteoblasts and participates in the signaling of downstream NF-κB receptor activating factor [37]. It is noted that hotairm1 inhibits RANKL-induced osteoclast formation through NF-κB pathway [50]. In addition, dysregulation of NF-κB is associated with several diseases that cause osteolysis, including periodontitis [51,52]. Herein, we found that the expression of AKT and NF-κB was significantly decreased after over expression of KLF10. When KLF10 was knocked down, the expression of AKT and NF-κB was significantly up-regulated. The addition of insulin reversed the expression trend of these factors. It is indicated that knockdown of KLF10 in insulin resistance may promote osteoblast differentiation and bone healing.

## Conclusion

In summary, our study revealed that KLF10 reduced the proliferation and differentiation of osteoblasts and decreased the expression of bone metabolism-related molecules and inhibited AKT and NF-κB pathways, whereas insulin reversed this effect after knockdown of KLF10. Knockdown of KLF10 in insulin resistance could promote osteoblast differentiation and dental implant osseointegration in diabetic patients. However, there are some limitations to our study. Firstly, the function of KLF10 in osteoclasts is needed to investigate in the further study. Secondly, the deeper molecular mechanism of KLF10 in bone metabolism is needed to further investigate.

## Supplementary Material

Supplemental Material

## Data Availability

All data are available in the article.
